# Sacred Foe: About the Face of Exemplary Evil

**DOI:** 10.1007/s11196-023-10077-0

**Published:** 2024-01-04

**Authors:** Massimo Leone

**Affiliations:** University of Turin, Via S. Anselmo 8, 10125 Turin, Italy

**Keywords:** Cain, Face, Mark, Semiotics, Foe, Evil

## Abstract

This essay aims to summarize and explore two issues that, in the exegetical and representational traditions of the biblical text, have triggered a myriad of semiotic intelligences. First, the nature of Cain’s face at the moment of the sacrifice refused him by the Lord, a face variously interpreted as angry, sad, dejected, depressed, dark. Second, the nature of the sign imposed by God on Cain following Abel’s fratricide. After exploring Jewish and Christian exegesis, ancient and modern, with some reference to contemporary narrative versions (and especially to Saramago’s *Cain*), the reflection will turn to the question of whether this kind of exegetical questioning can be part of the objects of a discipline like semiotics, the modern science of signs.

## Introduction

In a recent panel organized by Jenny Ponzo and her team at the 23rd roundtable for the semiotics of law (Rome, 24–27 May 2023), speakers were encouraged to reflect on “The role of exemplary characters in the interreligious translation of norms and religious practices”, in the framework of ERC project NeMoSanctI, led by Jenny Ponzo herself.^1^ In a nutshell, the panel underlined that the translation of a normative system encompassing behavioral codes, values, and beliefs from one culture to another presents a significant challenge in our increasingly globalized world. This issue becomes particularly complex when considering the subjectivity inherent in religious contexts. In intercultural encounters, mutual understanding can be facilitated when abstract norms undergo a process of figurativization, taking on tangible forms embodied by exemplary characters. Indeed, heroes, leaders, saints, and other symbolic or sacred figures within various cultures often serve as representations of social, moral, and religious systems and as models for behavior. They have the power to render abstract notions more accessible by weaving them into narrative structures, essentially telling a story. By giving these abstract principles a human shape, they evoke responses that go beyond mere intellectual understanding, delving into the realm of emotion. As a result, these exemplary figures become potent tools for communication. They possess the ability to impact all layers of society with remarkable efficiency. Yet the panel would equally prompt to acknowledge that these figures have frequently been sources of debate and conflict among proponents of different cultures or belief systems. Dialogues or conflicts surrounding these key figures carry significant consequences for how spaces related to their memory and worship are imbued with meaning. This, in turn, influences public and religious practices associated with such spaces. Sacred locations and “places of memory” emerge as pivotal settings where normative principles and ideas are shared, contested, or negotiated between diverse groups. Therefore, the panel would aim to focus on the role of exemplary characters in the context of intercultural and interreligious dialogues and in religious practices. The purpose was do so by presenting a diverse array of case studies, with a particular emphasis on exploring the representation and significance of Christian and Catholic figures within the dynamics of intercultural encounters.

In the same International Roundtable, a panel organized by the author of the present paper in his quality of director of the Center for Religious Studies at the Bruno Kessler Foundation in Trento^2^ promoted a reflection on “Dis-Embodiment in Religion, Ethics, and Law”. The panel would take as a point of departure the fact that all belief systems, and in particular those that are categorized as religions, fit into a dialectical field in which the sign manifestation can be posited as additive, subtractive, multiplicative, or divisive (to adopt a mildly mathematical metaphor and metalanguage). Additive manifestation takes place when a belief system increases its possibilities of signification, both immanent and transcendent, both internal and external, through the adoption of a signifying materiality, which is expressed in artefacts that are semiotically texts of culture. Subtractive manifestation, on the other hand, takes place when a belief system delineates or reinforces the perimeter of its own religious agency not by fabricating sacred signs, but by negating the mundane ones, i.e., those of other religions and belief systems. The multiplication of materiality can then take place through different modes of hybridization between the sign manifestations of religions, modes that include in the first instance those between different belief systems, resulting in syncretisms, but also those that interpenetrate the religious signification with the secular one. Finally, participants were also instigated to remember that the construction of a religious signification is often divisive, in the sense that it constructs the sacred in its deepest etymological and phenomenological sense, as separation from an otherness, but also because, in its manifestation, it designates not only the profane but also the heretical, the schismatic, and the abject.

This second panel’s aim was therefore to put this mathematics of religious expression to the test of the new forms of embodiment and disembodiment that are being elaborated within an increasingly global and digital infrastructure of religious communication, as well as in the contingency of a reformulation of community signification due to various factors and impediments, from pandemics to conflicts, from migrations to religiously motivated persecutions. New sign manifestations of the religious emerge from this problematic context of a simultaneous widening and narrowing of communication possibilities, often breaking the mold of the traditional combinatorics of religious expression. Believers and communities either become more visible thanks to the digital, or they disappear because they are unable to catch the wind of the new expressions. In all this whirlwind of new trends in religious signification, tensions and conflicts arise that in part exacerbate those of the pre-digital past, while in part resolve them, or dampen them, in a legal framework that struggles to formalize the new syncretisms and hybridities of digital religion.

The article that follows originates at the crossroad of the two panels described above, interweaving the three realms of the religious, the digital, and the legal. The key question at stake is the following: if religions construct and promote exemplary and embodied subjectivity, what is the role of the foe, in these systems of beliefs, subjectivations, and incorporations? Of the exemplary abject subject? And through what semiotics does this abjection manifest itself as the epitome of what religious cultures reject? In introducing the general subject of the Roundtable (“Global Semiotics and Everyday Legal Claims: Intercultural Use of Law, Interreligious Dialogue, and Translation Ethics”), Mario Ricca and Anne Wagner would emphasize that the relationship between legal rules and the spaces in which they are applied is undergoing a significant transformation, and this shift is not solely driven by political factors but also by semantic and cognitive considerations. The meaning of legal rules is continuously challenged as they adapt to changing spatial contexts and cultural frameworks. It is no longer valid to assume that legal rules neatly align with specific spatial circuits or cultural backgrounds. Instead, various territorial contexts, even those far removed from urban centers, can potentially serve as hubs for a multitude of actions and interests. These connections have a profound impact on the interpretation and effectiveness of legal rules, resulting in a state of spatial-semantic flux that permeates the everyday workings of the legal system.

The dynamics of contemporary law involve the convergence of diverse spatial and semantic dimensions, merging different ‘elsewheres’ and giving social phenomena and their legal regulation a form of ubiquity, at least in potential terms. This means that the traditional notions of ‘here’ and ‘now’ must—Wagner and Ricca would propose—be liberated from rigid associations with empirical events, objects, or situations. Instead, to truly understand the underlying phenomenality of these events/objects/situations and the implications of applying one legal rule over another, each should be viewed as a sign. Adopting a semiotic perspective enables the reconfiguration of the meaningful connections that underlie what we commonly label as ‘things’ and ‘events’, aligning them with the new scale of spatial implications resulting from multi-sited and global determinants affecting each local ‘here’.

Therefore, in the rationale of the roundtable, the intertwining of multiple ‘elsewheres’ necessitates a global semiotic comprehension that makes legal interpreters (and even lawmakers) aware of the semantic and spatial web that underpins any ‘fact’ to be adjudicated. The ability to grasp the strands of meaning within what is perceived as a ‘fact’ is also crucial for envisioning the consequences of applying specific legal rules and assessing the legitimacy of such applications within their axiological and teleological contexts. This presupposes an effort to translate the experiential dimensions from ‘other’ spaces implied in understanding the ‘present facts’ to be ruled upon, followed by an intercultural translation across different experiential realms entailed in this understanding. Moreover, since culture often encapsulates the anthropological and historical projections of religious meanings, any endeavor to facilitate intercultural translations intersects with the promotion of interreligious dialogue. Conversely, anthropological frameworks rooted in religious imagery shape even secularized experiential spaces and the related categorical structures that individuals use to define them. From this perspective, spatial and semantic Otherness should be seen—the Roundtable organizers would suggest—as an inherent element already involved in shaping people’s daily experiences. In this sense, transcending any rigid cultural identities, the capacity to recognize the semantic and pragmatic connections between physically distant elements can be significantly enhanced by engaging with experiential elements as signs that can be reconfigured and aggregated within new categorical frameworks.

The objective of the roundtable was, as a consequence, to bring together experts from various fields, including semiotics, anthropology, geography, law theory, and legal practice, encompassing civil law, business law, family law, international law, and legal anthropology. The intended aim was to demonstrate how a semiotic approach can serve as a potent tool for addressing the current challenges in legal practice and transforming it into a platform for emancipatory and bottom-up intercultural legal use.

To summarize what emerges as intersection of this general aim with the two panels described above, the roundtable became a place for discussing the challenges of translating normative systems across cultures, particularly in religious contexts, emphasizing the role of exemplary characters and examining the impact of evolving spatial and semantic dimensions in the globalized world.

The essay that follows, as it was suggested earlier, explores the extreme frontiers of this topology of exemplification, embodiment, and translation. It deals, in a way, with the untranslatable. It focuses on the body, and in particular on the face, when it becomes the symbol of the exemplary evil. It dwells on the multiple and paradoxical ways in which the untranslatable face of the foe becomes the necessary counterpart of a religious civilization’s inner topology, indicating what must be abject and rejected so that religious meaning can be introjected.

## A Dysphoric Face

“And Cain was very wroth, and his countenance fell” (Gen. 4:5), translates the KJV; “And Cain was very angry, and his countenance fell” (NKJV); “This made Cain very angry, and he looked dejected” (NLT); “So Cain was very angry, and his face was downcast” (NIV); “So Cain was very angry, and his face fell” (ESV); “Cain was furious, and he looked despondent” (CSB); […]; “iratusque est Cain vehementer et concidit vultus eius” (VUL); “καὶ ἐλύπησεν τὸν Καιν λίαν καὶ συνέπεσεν τῷ προσώπῳ” (LXX); “”, is the Masoretic text.

Gruber [3; 4] interprets the dejected face as an expression of depression:

Cain’s reaction, according to most English translations, was anger. The New English Bible tells us, “Cain was very angry and his face fell”. In the cadenced measures of the traditional King James version, we find “And Cain was very wroth, and his countenance fell”. I believe that this is a mistranslation. The emotion which the Bible records here is depression not anger. The story of Cain and Abel is the first depressive episode mentioned in the Bible. This first case of depression led to the first Biblical murder.

[4: 34]

But the expression, idiomatically, is used only one other time, in Jeremiah 3:12, which the KJV translates as “and I will not cause mine anger to fall upon you” and the NIV as “I will frown on you no longer”; “frowning face” for the New Revised; “et non avertam faciem meam a vobis” (VUL); “” (LXX). The literature seems to lean toward anger, rather than “depression” or “abasement” (which instead appears in some English translations); Kruger [6] and Schlimm [14] opt for wrath. But does it really make sense to channel biblical semantics into that of contemporary emotions? No matter how one answers it, identifying the emotion of the face (outraged or dejected) implies relevant interpretive consequences, not only of the biblical passage but also of its immense cultural legacy.

In fact, translating the passage is not only a matter of semantics but also one of syntax. The Septuagint translates Gen 4:5 “καὶ συνέπεσεν τῷ προσώπῳ”; the ancient Greek lacked the same idiomatic formula, but the translator also chose to omit the possessive and used the dative (“and he fell down with respect to the face,” one might translate in English). According to Wevers [23: 51] “by using the dative ‘he fell together with respect to the face’ he places the emphasis on Cain.” In the Septuagint, in short, as Greimasian semiotics would put it, the use of the dative focuses the attention of the observing actant on Cain, as if he himself fell or collapsed completely together with respect to his own dejected face. The Vulgate seems to trace this total falling of Cain with his face, translating “cur concidit facies tua.” Each new version adds semantic nuances in the exercise of translating, but also in the attempt to explain by translating.

The Syriac versions introduce a further variation: the face of Cain is not “cast down” but “darkened”:  [9: 73]. According to Scarlata [13: 58], “The association of Cain with Samma’el (i.e. the Satan), and the tradition that Saturn is the Star of Evil that brings calamity upon Israel, is what gave rise to the word play in Hebrew exegesis which interpreted the biblical *qyn* (Cain) as *kywn* (Saturn), and *wyplw ppgyw* (“and his face fell”) as *wy’plw pnyw* (“and his face darkened”).” Luis Ginzberg comments on this issue in the first volume of *The Legends of the Jews* [1, 2: 101]: “But after the fall of Eve, Satan, in the guise of the serpent, approached her, and the fruit of their union was Cain, the ancestor of all the impious generations that were rebellious toward God, and rose up against Him.”

Ginzberg recalls that the *Vita Adae*^3^ identifies Cain as the son of the union between Eve and Samael, the Satanic archangel. According to Scholem [15: 385–388] “from the Amoraic period onward Samael is the major name of Satan in Judaism” [15: 385]. The great scholar of Jewish mysticism brings together the sources for this figure, whose name etymologically refers to blindness, , while the *Zohar Hadash* (31, 4) describes him as “dark in the face.” According to the *Vita Adae*, however, the son of Samael and Eve, i.e., Cain, is born neither blind nor dark; of his birth on the contrary, it is narrated in one version that Eve “peperit filium et erat lucidus”^4^; another version adds “erat que ut stella lucidus”.^5^ Is the brightness of Cain’s face already Luciferian, linked to an evil astronomy [1]? Ginzberg inclines toward this interpretation [1, 2: 103 n. 6], especially when he “corrects” the *Revelation of Moses*. In this text, in fact, we read about Cain what follows [18: 122]:
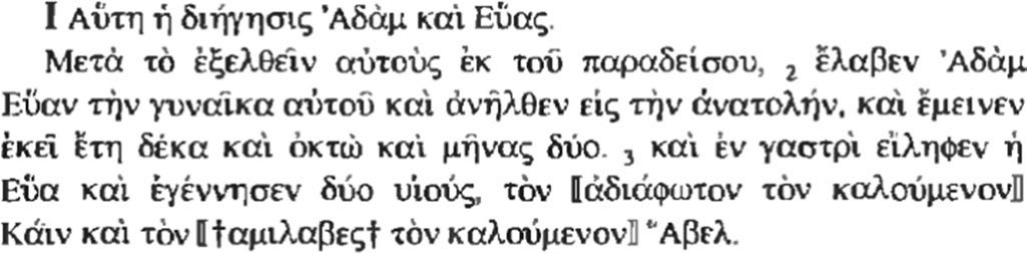


Cain is nicknamed , meaning literally he who (whose face) is not crossed by light. But Ginzberg [1, 2: 103 n. 6] believes it should read “διάφωτον”, not “ἀδιάφωτον,” contradicting the earlier philological lesson [17]:

Vita Adae 21, according to which Adiaphotus in Apocalypse of Moses 1 should be changed to Diaphotus, “full of light.” On Cain’s luminous countenance comp. PRE 21 (Eve saw that his countenance was heavenly) and Targum Yerushalmi Gen. 4. The similarity of Cain  to Kewan , “Saturn”, may have given rise to this legend about the shining countenance, particularly if one considers, on the one hand, the relationship between Cain and Sammael (=Satan [...]), and, on the other hand, the fact that Saturn represents the star of evil which brings misfortune to Israel.

In short, was Cain’s face at birth dark or bright? Crossed by light or dull? Lachs [7] attempts to shed light on the issue starting from the opposition between  and the way the same Greek text of the Apocalypse of Moses nicknames Abel, viz. .^6^ Since the Armenian version of the text names Abel “Anloys,” i.e., “with light”, Lachs prefers to retain the designation of Cain as , despite the fact that earlier authors had proposed different solutions; not only Ginzberg  but also the translator of the Greek text for the Ante-Nicene Christian Library (XVI, 1870), who proposes to change , meaning “the planter”, in the sense of “cultivator of plants”, to be opposed to , meaning “keeper of the flock”. Lachs instead retains , as he opposes it to a reading of  as a reference to the Masoretic text of Gen 3:21 , “the LORD God make coats of skins, and clothed them” (KJV), but transformed in the midrashic reception, which, with a play on words, mutates the skin into , “light.” Leaning on this literature, already cited and commented on by Ginzberg [1, 2: 80 n. 93], Lachs believes that the opposition between the nicknames of Cain and Abel refers to the contrast between the one whose face from birth is dark and devoid of light because he is the son of an evil angel and the one whose face is instead covered with light. Is this perhaps the final interpretive solution, contrary to that proposed by Ginzberg?

Philologists have been divided on the point for years. Tromp [18: 278 n. 7] lists the proponents of either position, but shows that, philologically, the variant  is to be considered archetypal in all Greek manuscripts, while  is a variant found in almost all manuscripts. Although these two terms are attested philologically, however, their genesis reveals a surprise. If one looks up  in the lexicons of ancient Greek, one finds nothing. In fact, the term is used only in the Greek versions of the lives of Adam and Eve. What is found is , “to illuminate”. In the seventh century, the historian Siror John Malalas (Ἰωάννης Μαλάλας, Iōánnēs Malálas) uses this verb to describe a grand illuminated palace.

One of the most recent philological hypotheses, expounded by Tromp [18], is that this term is a neologism created to designate Cain as an “unenlightened”, i.e., obscure being, as opposed to Abel’s nickname,  which, however, would result from a textual transcription error. To sum up: a dictography unintentionally “invents” for Abel the nickname , which means nothing at least in the intention of the person making the error. Then to follow, the nickname  is given to Cain, to contrast it with that of Abel, whose second accidental name in the meantime, however, had been interpreted in relation to the semantic sphere of light, to the clothes of light in the midrashic interpretation, or to light in the Armenian versions. By contrast, Cain is designated as the one whose face is devoid of light. But this designation conflicts with other texts, such as the Latin Life of Adam and Eve, which instead emphasizes that, at birth, Cain was luminous, but of a luminosity often interpreted as already luciferous, that is, attributable to Cain’s own satanic parent. On the other hand, however, other traditions link Cain’s name to the blindness and darkness inherent in the etymology of his father’s name Samael, that is, to a saturnine, malefic, negative luminosity. This perhaps explains why the Syriac versions of the story of Cain and Abel, in translating Gen. 4:5, designate the former’s face as “darkened”, and not as “angry” or “downcast.”

But it is perhaps inappropriate to search the jungle of biblical versions and their interpretations for systematic coherence. Instead, one finds there rhizomatic tensions that create, as contemporary semiotics would say, semi-symbolic systems, in which Abel is pitted against Cain according to a logic reminiscent of that found by Roman Jakobson in his famous article on affirmative and negative gestures [5]. Whether one reads Cain’s face as “luminescent” or as “devoid of light”, the important thing is that this luminous quality of his is contrasted with that of his brother Abel, either because the former, unlike the latter, is not clothed in the “robes of light” bestowed on Adam and Eve, and is instead livid in the face from envy, anger or despondency, or because, again in contrast to Abel, he is the son of an evil, nocturnal being, of a satanic angel whose name refers to blindness and darkness.

Yet Cain’s face is not interpreted as dark only because it is dejected, or because it lacks the light that emanates from his brother’s face, or because it is the legacy of a satanic lineage, but also according to the precise circumstances that imprint this darkness on it. His reaction, which contemporary semiotics would call “dysphoric”, is again contrasted with his brother’s “euphoric” one. The Masoretic text does not explain why on earth the former’s sacrifice is rejected while the latter’s is accepted, but this omission sparks the interpretive imagination of later exegesis, which often revolves around the relationship between sacrifice and face. Something is wrong with Cain’s sacrifice. Ginzberg [1, 2: 103–4] summarizes the interpretations: because whereas Abel had chosen to sacrifice the best part of his livestock, Cain first ate himself, and then sacrificed only a few flax seeds; because Abel had sacrificed all his offerings, whereas Cain had kept a portion of them for himself.

According to other interpretations, however, the issue was not so much the object of the sacrifice as its manner. Abel had sacrificed modestly, while Cain, according to the *Zohar Hadash* (24a, on Gen. 4:2), had sacrificed haughtily, without that contrite spirit that the Psalms recommend (Psa. 51:19). But there are also those who ascribe Abel’s tragic end to the fact that he, in sacrificing, had overlooked the face of the Lord; Abel’s imprudent gaze is thus related to the prudent gaze of Moses, who will treasure the tragic affair of the first man killed in Ex. 3: 6 [19, 20]. Abel therefore dies because he stares into the face of the Lord, but in contrast Cain’s face darkens because his sacrificial offerings are deemed unworthy and, therefore, rejected. The smoke that is supposed to consume them fails to rise to the heavens and then turns back to Cain himself, whose face is darkened not only because of envy, anger, sadness, and humiliation (in other traditions, his failure to attempt the sacrifice is greeted with mockery by his family), but also because the smoke of the sacrifice returns to his face.

## A Scarred Face

Abel tinha o seu gado, caim o seu agro, e, como mandavam a tradição e a obrigação religiosa, ofereceram ao senhor as primícias do seu trabalho, queimando abel a delicada carne de um cordeiro e caim os produtos da terra, umas quantas espigas e sementes. Sucedeu então algo até hoje inexplicado.

[12]^7^

In this way José Saramago begins to recount the episode of the parallel sacrifice by Abel and Cain in one of his last novels, *Caim* (2009), evidently drawing on apocryphal literature, and reporting the circumstances of the smoke rising mightily from Abel’s animal sacrifice, while Cain’s plant sacrifice is rejected:

Sucedeu então algo até hoje inexplicado. O fumo da carne oferecida por abel subiu a direito até desaparecer no espaço infinito, sinal de que o senhor aceitava o sacrifício e nele se comprazia, mas o fumo dos vegetais de caim, cultivados com um amor pelo menos igual, não foi longe, dispersou-se logo ali, a pouca altura do solo, o que significava que o senhor o rejeitava sem qualquer contemplação.

(*Ibidem*)^8^

Saramago points out that the rejection of Cain’s sacrifice remains to this day without explanation; the Portuguese writer then goes on, with habitual irreverence,^9^ to retell the biblical episode, narrating the killing of Abel, but also inventing a seditious Cain, who instead of passively accepting divine punishment counterattacks and accuses his creator of being co-responsible for Abel’s death. Even this invention, however, rests, as it is often the case in the novels of the Portuguese Nobel laureate, on apocryphal literature (and probably on the compendium of it provided by [2], 1: 108 n. 20). This iconoclastic version of the tale is one of the last ones, but comes after a very long tradition of exegetical, narrative, and iconographic reworkings of the episode, which often focus on Cain’s face. The element that most attracts the imagination of those who elaborate the biblical text, however, is obviously Gen 4:15: “And the LORD set a mark upon Cain, lest any finding him should kill him” (KJV); “posuitque Dominus Cain signum ut non eum interficeret omnis qui invenisset eum” (VUL);  (New Diodates).

On the pragmatics of this sign, that is, its intended effects in the context in which its reception could have taken place, the debate is heated, but guided by the biblical text itself, which describes them precisely. The sign was to immediately distinguish Cain in his wandering among other men, and to ensure that they would not strike or kill him. From this we deduce not only the obvious fact that other men did not bear the same sign on themselves, but also the more interesting fact that it was such as to have an immediate pragmatic effect on other human beings. Anyone who would have seen it would not have struck or killed Cain. On the one hand, such pragmatic logic is to be linked to the punishment inflicted on the first murderer: destined to wander in a world of settled people, he is also condemned to traverse alien lands perpetually (or at least until his death, or the fulfillment of his own punishment, according to some interpretations), and thus to be perceived as an invader, as a danger. On the other hand, exegetes debate the moral nature of this pragmatics, that is, whether it is intended to protect Cain, to protect his punishment from premature termination, or both. In this regard, Westermann [22: 312] claims what follows:

It is clearly stated and leaves no ground for misunderstanding that the mark is to protect Cain the cursed and the outcast from being a prey to other people. The mark is linked indissolubly with the legal ordinance whose function is just this. The conclusion is that the mark can only have an individual meaning and not a collective one.

A real interpretive chasm opens, however, when the exegete tries to explain the origin of this pragmatics, that is, from what syntactic arrangement and semantic content a sign affixed to Cain could receive such symbolic force. If one looks at the question, that is, at the syntax and semantics of the Cain sign, through the eyes of contemporary semiotics, the question cannot but be absurd. For no sign, however it is composed or received, could protect in this way, subject as it would be to all the interpretive variables with which contemporary semiotics is well acquainted. For the latter, indeed, the sign of Cain can only be configured as a “magical” sign, in the sense that its pragmatic efficacy is due to those who believe in it, that is, to the prodigy of those who created it, and not to its syntactic or semantic structure. In short, for contemporary semiotics the sign of Cain would be effective only for those who already knew it and adhered to it, that is, by virtue of a supernatural, extra-semiotic power.

But to read ancient exegetical discourses through the eyes of the modern discipline of signs would be trivializing; in fact, what is most interesting is how, through the centuries, interpreters of all cultures and genres have sought to explore this chasm of interpretation. Indeed, contemporary semiotics must register that such an exegetical chasm constitutes in itself an interesting object of meta-reflection on sign cultures and their interpretation. Not only in the Jewish and then Christian and Islamic traditions, but perhaps in all cultures in general, whenever a sign is attributed total and even dramatic pragmatic efficacy (Cain removed from death at the hands of others),^10^ but without explaining how this happens, the signs that produce the pragmatic effect become a secret that irresistibly attracts the curiosity of exegetes.

This is also the case with the mysterious “sign of Cain”. All modern commentators point this out: “What this sign, or mark, consisted of, and how it should be construed, has been debated for centuries” [21]; “The nature of Cain’s sign or mark has been the subject of endless inconclusive speculation” [13: 180]. While exegetes try to cross the interpretive crevasse with pure speculation, apocryphal texts fill it narratively: God inscribes a letter of his own name on Cain’s forehead; he also addresses the animals, admonishing them that Cain’s punishment should not be like that of future murderers, since he killed without knowing the consequences of his actions, being precisely the first murderer; God then assigns Cain the dog as protection from the other beasts, and marks not only the face but also the body of the fratricide, with leprosy [2], 1: 107].

*Bereshit Rabba* (*Midrash Rabbah* 1: 22: 12–13) [10] proposes as usual different interpretations: R. Judah believes that the sign consists in “the solar globe shining for him”; to which R. Nehemiah contests, “For that ruin to make the solar globe shine!”; this last interpreter therefore proposes that the sign is leprosy (based on Ex 4: 8: “And it shall come to pass, if they will not believe thee, neither hearken to the voice of the first sign, that they will believe the voice of the latter sign” (KJV), where the “first sign” is the one evoked in verse 6, i.e., precisely leprosy: “And the LORD said furthermore unto him, Put now thine hand into thy bosom. And he put his hand into his bosom: and when he took it out, behold, his hand was leprous as snow” (KJV). According to Rab, the sign is the dog; according to Abba Jose, a horn growing out of Cain’s body; Rab then proposes an interesting commentary, also based on the syntax of the biblical verse: the Lord imposes a sign on Cain, but he himself becomes a sign for the other murderers, thus triggering a semiosis that runs parallel to his punishment. According to R. Ḥanin, Cain is made a sign, however, not for the murderers but for the penitents. R. Levi, speaking on behalf of R. Simeon B. Laḳish, suggests that “He [the Lord] suspended His judgment until the flood came […]”. The *Tanhuma Yelamdenu* adds an eighth interpretation: the Shabbat, a sign between the Lord and man, came to the salvation of the latter, as had already happened with Adam.

In his *Excursus* on the sign of Cain [22: 312–315], Westermann divides exegesis on the subject into five groups, according to how they interpret the verse “The Lord imposed on Cain a sign”; groups are identified depending on whether this signification concerns an individual or a collectivity; according to the function of the sign, either identifying or protective; according to the form of the sign; and according to whether or not they are willing to propose amendments to the text. Bernhard Stade devotes a long essay [16] to the subject, in which he compares this “stigma” with those that other cultures impose on offenders. He emphasizes that body deformation is the only mnemotechnique that can permanently associate a message with an individual: “Der Mensch hat kein anderes Mittel, ein bleibendes Zeichen an sich hervorzubringen, als die Deformation seines Körpers” [16: 301–2].^11^ With this anthropological perspective, the author thus mentions several instances where cultures deform bodies by various means and results, either to punish, to admonish, or to protect, which seems to be the function of the sign imposed by the Lord on Cain.

Some of these signs concern the face; indeed, the biblical text does not make explicit which part of Cain’s body is deformed by the divine sign; some exegetes, as we have seen, even lean toward “external” signs, such as the shining sun or the dog. However, these latter interpretations are unconvincing: the idea of a sign accompanying Cain without his being able to separate from it, and without the sign being able to separate itself from Cain, is more in keeping with the narrative structure of the episode. It is for this reason, as well as to emphasize the visibility of the sign, that many interpreters have thought of the face, and even the forehead — although, it is worth repeating, the biblical text is not explicit in this regard. Stade cites as an example the mark of infamy imposed on the swindler, and especially the forger, in pre-modern legal conceptions: “Der Gauner, der seine Genossen verrathen hatte, wurde von diesen durch den *Slichenerzinken* [italics in text], einen Schnitt in die Wange oder eine andere Deformation des Gesichtes, als Verrther kenntlich gemacht” [16: 251].^12^

In a pamphlet published in 1713, the theologian Christian Karl Stempel had already summarized all the ancient hypotheses, including those who believed that the sign consisted of an engraved or tattooed letter^13^ on Cain’s forehead [16: 9]:

*Rabbi Eliezer in Pirke c. 21.* itemque *R. Salomon Iarchi* quem allegat Ottho Lex. Rabb. Phil. p. 98. conjiciunt, ſignum hoc fuiſſe literam ex nomine tetragrammato, forſan, vel ex nomine * poenitentia*, ut quidam Hebraeorum aiunt, vel ut *Paulus Fagius* vult, citante *Matth. Polo in Synopſ. Crit.Vol. 1.* ex ipſo Caini nomine fronti vel brachio inſculptam, ut eo omnes facile admonerentur, Cainum poenituiffe, ideoque ei parcerent.^14^

“Fronti vel brachio insculptam,” “a letter inscribed on the forehead or arm”. The forehead is also the place where, according to another translation, the horn marking Cain’s face would sprout, a horn whose function is multiple: to stigmatize the fratricide forever; but also to act as a deterrent against anyone who would want to kill him, thus fulfilling the dual function of a sign of infamy and protection: “Similem huic plane fentiam apud eundem *Pfeifferum* 1.c. fouet *Abba loſe ben Kiſri in Zennor*, qui cornu e fronte Caini enatum comminiſcitur” [16: 8].^15^

In the face of such a proliferation of assumptions, contemporary biblical scholarship is categorical:

I agree with those scholars who refuse to give any answer to this question. This refusal can be justified; we are dealing here with a primeval narrative. This means in the present case that the narrator is dealing with an event that is beyond the present, where things happen differently from the world of time with which we are familiar. He did not mean a mark familiar and demonstrable to his contemporaries; he had no interest at all how this mark was to be presented. It has meaning only in the context which the narrative intends to describe. We must acknowledge that even the narrator himself had no definite idea of the form of the sign.

[22: 314]

## Conclusions

What did Cain’s face look like at birth? Luminescent or dark? And what similarities did it show? Did it really resemble the face of a rebellious angel? And in the face of the Lord’s denial, did this human face show depressed, sad, dejected, or angry, or livid with envy at his brother and his grateful sacrifice to God? And once the fratricide was accomplished, what sign did the Lord impose on Cain, where, how, and for what purpose? Or was it Cain himself who was transformed into a sign? Semiotics, the modern discipline of signs, as well as other contemporary text methodologies, cannot answer such questions, nor are they supposed to ask them. Yet it is not recommendable either that text analysis irreverently disdains all the questions that generations of commentators and exegetes have addressed with respect to the face of Cain, and the sign imprinted on it by divine will. The semiotics of the text can try to go along with the intentionality of the structure that weaves the divine narrative, knowing full well that it is an ancient tale, made up of remote words, translated multiple times, multiple times corrupted. But there is also room for a semiotics of biblical cultures, which is not so much interested in choosing among the answers that exegetes have been giving to their questions for millennia, but in collecting both of them, both questions and solutions, into a reasoned typology which shows how, around the ‘crevices of meaning’ opened in the biblical textual tradition, each approach has tried to fill the chasm according to different mindsets, which reveal much about the way in which this millennial text and its endless tales have been used from time to time to construct the meaning not only and not so much of the narrative but of the storytelling community, of its way of understanding signs, meaning, face, and the sacred.

## Footnotes

1 https://nemosancti.eu/.

2 https://isr.fbk.eu/en/.

3 *Vita Adae et Euae sec. recensionem lat-P*, 21, 3a, 8, p. 327 (Brepols: Library of Latin Text; available at http://clt.brepolis.net/llta/pages/Toc.aspx?ctx=5929555).

4 “She bore a son and he was bright”; *Vita Adae et Euae sec. recensionem lat-V*, 21, 3a, 7, p. 326.

5 “And it was bright as a star”; *Vita Adae et Euae sec. recensionem lat-P*, 21, 3a, 7, p. 326.

6 That is, “Amilabes,” Abel’s biblical nickname.

7 “Abel had his cattle, Cain his crops, and, as tradition and religious obligation required, they offered to the Lord the first fruits of their labor, Abel by burning the delicate meat of a lamb and Cain the produce of the earth, a few ears of corn and seeds. Then something happened that, to this day, remains unexplained”; transl. mine.

8 “Then something inexplicable happened. The smoke from the meat offered by Abel went straight up until it disappeared into infinite space, a sign that the Lord accepted the sacrifice and was pleased with it, but the smoke from Cain’s vegetables, cultivated with at least as much love, did not go far, it dissipated right there, a little above the earth, which means that the Lord rejected it without any qualms”; transl. mine.

9 Modern reinterpretations that seek to exonerate Cain are countless: Hugo, Unamuno, Hesse, Betti, Ungaretti, Gualtieri, Camilleri, to name a few.

10 But think also of the mysterious signs drawn by Jesus in the pericope of the Adulteress, which again seem to help save her from stoning but about which we are not told how they were made or what they meant, again opening a chasm of possible exegesis [8].

11 “Man has no other means of producing a permanent mark in himself than the deformation of his own body”; transl. mine.

12 “The thug who had betrayed his comrades was identified as a traitor by the disfigurement, a cut in the cheek or other deformation of the face”; trans. mine.

13 Migliore 2018 [11: 44 n. 12] links the Jewish and later Christian ban on tattooing to the Cain episode; however, the issue is more complex: first, because as we have seen, the interpretation of the sign of Cain as a tattoo is not unanimous in the Jewish world; second, because the practice of tattooing, and its ban in *Leviticus*, could instead be attributed to a desire not to subject the Jews’ bodies to practices seen as “foreign”, and therefore idolatrous.

14 *Rabbi Eliezer* in *Pirke*, c. 21, and similarly *R. Salomon Iarchi*, who cites Ottho Lex. Rabb. Phil. p. 98, conjecture that such a sign was a letter from the tetragrammatical name, or perhaps one from the word , *penance*, as claimed by some among the Jews, or, as *Paulus Fagius*, who cites *Matth. Pole in Synopſ. Crit.Vol. 1.*, from the same name of Cain, engraved on the forehead or arm, so that by this all might easily be admonished, that Cain was a penitent, and should for that very reason be spared”; transl. mine.

15 “Similar explanation is found at the same *Pfeifferum* 1.c. in support of *Abba lose ben Kisri in Zennor*, who conjectures that a horn sprouted from Cain’s forehead”; transl. mine.
